# Endocrine disorders in Beta-Thalassemia major patients at a Tertiary Care Hospital

**DOI:** 10.12669/pjms.39.3.6837

**Published:** 2023

**Authors:** Suleman Elahi Malik, Shaista Kanwal, Javeria Javed, Wagma Hidayat, Tahir Ghaffar, Azizul Hasan Aamir

**Affiliations:** 1 Dr. Suleman Elahi Malik, MBBS, FCPS Medicine, FCPS Endocrinology. Endocrinology Division, Department of Medical Specialties, MTI Khyber Teaching Hospital, Peshawar, Pakistan.; 2 Dr. Shaista Kanwal, MBBS, FCPS Medicine, FCPS Endocrinology, MRCP (UK). Department of Diabetes, Endocrinology & Metabolic Diseases, MTI, Hayatabad Medical Complex, Peshawar, Pakistan; 3 Dr. Javeria Javed, MBBS. Department of Diabetes, Endocrinology & Metabolic Diseases, MTI, Hayatabad Medical Complex, Peshawar, Pakistan; 4 Dr. Wagma Hidayat, MBBS. Department of Diabetes, Endocrinology & Metabolic Diseases, MTI, Hayatabad Medical Complex, Peshawar, Pakistan; 5 Dr. Tahir Ghaffar, MBBS, FCPS Endocrinology, FCPS Medicine, MRCP (UK). Department of Diabetes, Endocrinology & Metabolic Diseases, MTI, Hayatabad Medical Complex, Peshawar, Pakistan; 6 Dr. Azizul Hasan Aamir, MRCP (UK), FRCP (Edin), FACE (US). Department of Diabetes, Endocrinology & Metabolic Diseases, MTI, Hayatabad Medical Complex, Peshawar, Pakistan

**Keywords:** Endocrine Disorders, Beta-Thalassemia Major, Short Stature, Thyroid Dysfunctions, Diabetes Mellitus, Parathyroid Dysfunctions, Pituitary Dysfunctions, Tanner Staging

## Abstract

**Objectives:**

To determine the frequency of endocrine disorders in Beta-Thalassemia Major (BTM) patients presenting for Endocrine Evaluation to the Department of Diabetes, Endocrinology and Metabolic Diseases, Hayatabad Medical Complex, Peshawar, Pakistan, a tertiary care hospital.

**Method:**

This descriptive study was conducted in the Department of Diabetes, Endocrinology and Metabolic Diseases, Hayatabad Medical Complex, Peshawar from October 2019 to August 2021. All patients with BTM presenting for endocrine evaluation were included in the study. Height and weight were assessed and plotted on the standard charts. For secondary sexual characteristics tanner staging was used. Blood samples for hormonal profile were taken according to standard protocol and sent for endocrine assessment.

**Results:**

A Total of 135 patients BTM were enrolled in the study comprising of 70 (51.9%) males and 65 (48.1%) females. Their mean age was 14.8±3.9 years, mean height 138.5±13.01 cm, mean weight 35.9±8.4 kg, mean BMI 18.6±2.8 kg/m^2^, mean age of transfusion started was 6.7±3.99 months, mean duration of transfusion 13.6±4.03 years and mean duration of chelation therapy received 6.1±4.5 years. Regarding endocrine complications, out of 135 patients assessed, one hundred (74.1%) had height less than 5^th^ centile and fifteen (11.1%) had diabetes mellitus. For thyroid and parathyroid function, 58 and 13 were tested respectively, out of which 16 (27.6%) and 6 (46.2%) had thyroid dysfunction and hypoparathyroidism. Out of 91 patients assessed for pubertal delay, 61 (67.03%) had delayed puberty.

**Conclusions:**

High percentage of endocrine complications were found in patients with BTM. Severity and multiplicity of endocrine organs involvement was dependent on duration of the disease and lack of compliance with chelation therapy.

## INTRODUCTION

Beta thalassemia is an autosomal recessive inherited disorder characterized by defect in beta chain synthesis which can either be homozygous or heterozygous. In homozygous state it is referred as Beta thalassemia major (BTM) while in heterozygous state it is known as thalassemia minor. BTM is a lifelong blood transfusion dependent disorder.^1^ Regular blood transfusions are required in BTM patients to maintain hemoglobin levels. This leads to secondary iron overload and its deposition, which is treated with iron chelation therapy.^2^ Excessive iron deposition in multiple organs including liver, joints, skin, heart, and endocrine glands leads to complications like liver cirrhosis, arthritis, cardiomyopathy, congestive heart failure and endocrinopathies, respectively.^3^ Endocrinopathies are one of the most common complications of transfusion dependent thalassemia (TDT) and includes hypopituitarism, hypothyroidism, hypoparathyroidism, hypogonadism (delay in puberty), impaired glucose tolerance (diabetes mellitus) and growth abnormalities (short stature).^4^

Beta thalassemia is prevalent in many countries, but the highest carriers are found in Cyprus (14%), Sardinia (10.3%) and Southeast Asia.^5^ Globally, it is estimated that around 80 million people are carriers for beta thalassemia disorder and around 23,000 babies are born with thalassemia major each year and amongst these, 90% belongs to low-middle income countries.^6^ In Pakistan Beta thalassemia is considered to be among the most common inherited class of hemoglobinopathies which is evident by the fact that around 9.8 million are carriers among the general population. According to an estimate, around 50000 BTM patients are currently registered in Pakistan and between 5000-9000 news cases per Anum are added to it, making Pakistan to stand in the list of country having highest prevalence of TDT patients.^7^

Endocrine complications are the commonest among BTM patients and the attributing factor is iron deposition in endocrine organs due to frequent blood transfusions along with sub-optimal iron chelation. These endocrine complications adversely affect the quality of life and increase the burden of disease on patient both physically and financially.^8^ A study conducted in Karachi showed that short stature and delayed puberty were present in more than 70% of BTM patients.^9^ However, in this regard there is scarcity of data in our local region. Therefore, the aim of this study was to evaluate the BTM patients from the endocrine point of view to project the burden of endocrinopathies in BTM patients in our local region. It will also identify the most common endocrinopathies in our patients which will have an impact on their overall management like routine evaluation for endocrine complication at least yearly. This will also emphasize the importance of early chelation therapy and compliance to it. Routine evaluation of BTM patients for endocrinopathies and their proper management will improve quality of life of these patients who are already fighting with lifelong illness.

## METHODS

This descriptive study of 22 months duration was conducted in the Department of Diabetes, Endocrinology and Metabolic Diseases, Hayatabad Medical Complex, Peshawar from October 2019 to August 2021. The study was approved by the institutional research and ethics committee under Ref. No. 230/HEC/B&PC dated 4^th^ October 2019. A written informed consent was obtained from all the patients enrolled in the study. Using non-probability consecutive sampling technique, BTM patients both male and female, who were on regular blood transfusion and age range from 10-30 years were enrolled in the study. After taking detailed history and physical examination, all the relevant information was recorded on a pre-designed questionnaire. Patients with acute kidney injury, sepsis, malignancies, critical illnesses, and those using calcium/Vitamin D supplements or under any treatment for endocrinopathies or with history of intake of traditional medications like herbal and hakeemi medications were excluded from the study.

Height and weight of the patients were plotted on the Centre for Disease Control and Prevention (CDC) 2000 age/gender specific growth charts and patients whose heights were below the 5^th^ percentile for their age were labelled as short stature. Secondary sexual characteristics were assessed and staged according to the Tanner staging system. Pubertal status was assessed in girls over 13 years and in boys over 14 years by Tanner’s classification. Delayed puberty was indicated by absence of breast development in girls by age 13 years and absence of testicular enlargement in boys (less than 4 ml) as measured by Prader’sorchidometer by the age of 14.^9^ The blood samples were taken according to standard protocol for analysis like Complete Blood Count (CBC), Serum Ferritin, Luteinizing Hormone (LH), Follicle stimulating hormone (FSH), Thyroid stimulating hormone (TSH), Free T4, Total T3, Intact Parathyroid hormone (PTH), Serum Calcium, Serum Phosphorus, Serum Testosterone & Blood Glucose by Roche Cobas 6000 analyzer series. All the patients were assessed for short stature, diabetes mellitus and pubertal development while only 58 and 13 patients were assessed for thyroid and parathyroid function, respectively. FSH and LH was measured in only those patients who had pubertal delay provided they could afford it. Moreover, relationship of endocrine complications with duration of transfusion and compliance to chelation therapy was also assessed.

For statistical analysis data was stored and analyzed by the statistical program SPSS version 19.0. Mean ± standard deviation was calculated for quantitative data whereas percentages were calculated for categorical data. The relationship of endocrine complications with the duration of transfusion and compliance to chelation therapy was determined by performing chi-square test. P-value of less than 0.05 was considered statistically significant. All the results are presented in tables accordingly.

## RESULTS

A total of 135 participants were enrolled for the study, of whom there were 70 (51.9%) males and 65 (48.1%) females. They had a mean age of 14.8 ± 3.9 years, mean height 138.5 ± 13.01 cm, mean weight 35.9 ± 8.4 kg and mean BMI 18.6 ± 2.8 kg/m^2^. Mean age of transfusion started was 6.7 ± 3.99 months, mean duration of transfusion was 13.6 ± 4.03 years and mean duration of chelation therapy received was 6.1 ± 4.5 years. Mean serum ferritin was 5219.94 ± 3076.3 ng/ml, mean FBS was 109.8 ± 56.97 mg/dl and mean TSH was 4.9 ± 4.3 uIU/ml. Mean serum calcium, phosphorus and iPTH were 9.04 ± 1.03 mg/dl, 4.7 ± 1.2 mg/dl and 25.4 ± 20.4 pg/ml respectively. Similarly mean LH, FSH and serum testosterone were 3.5 ± 3.8 mIU/ml, 3.6 ± 3.7 mIU/ml and 10.7 ± 54.4 ng/ml respectively.

Regarding endocrine complications, out of 135 patients assessed, one hundred (74.1%) had height less than 5th centile and fifteen (11.1%) had diabetes mellitus. Regarding gonadal function, out of 91 patients, sixty one (67.03%) had delayed puberty. For thyroid and parathyroid function, 58 and 13 were tested respectively, out of which 16 (27.6%) and six (46.2%) had thyroid dysfunction and hypoparathyroidism. Overall, 74.1% (28/135) of patients suffered from at least one endocrine complication and only 3.7% (5/135) of patients knew about the long-term benefits of chelation therapy. Short stature was the most common endocrinopathy while diabetes mellitus was the least common endocrinopathy. The frequency of endocrine complications is presented in Table-I.

**Table-I:** Frequency of endocrine complications in-patients with beta thalassemia major.

Endocrine Complications		Number of Patients	Percentage of Patients Among Assessed
Height centile	Less than 5^th^ centile	100	74.1
	5^th^ to 25^th^ centile	27	20.0
	25^th^ to 50^th^centile	5	3.7
	More than 50^th^ centile	3	2.2
	Total Assessed	135	
Thyroid Function	Euthyroidism	42	72.4
	Hypothyroidism	11	19.0
	Subclinical hypothyroidism	5	8.6
	Total Assessed	58	
Parathyroid function	Normal	7	53.8
	Hypoparathyroidism	6	46.2
	Total Assessed	13	9.6
Gonadal function	Normal puberty	30	32.97
	Delayed puberty	61	67.03
	Total Assessed	91	
Diabetes Mellitus	No	120	88.9
	Yes	15	11.1
	Total Assessed	135	

The relation of short stature, delayed puberty, diabetes mellitus, thyroid and parathyroid dysfunction with the duration of transfusion was assessed and is presented in Table-II. Hypoparathyroidism was more common in patients who had transfusion duration of more than 15 years, and this was statistically significant (p value= 0.03). Similarly, delayed puberty was also more frequently observed in patients with transfusion duration of more than 15 years and it was also statistically significant with p value of less than 0.0001. Likewise, the relation of endocrine complications with compliance to chelation therapy was also assessed and is presented in Table-III.

**Table-II:** Relationship between duration of transfusion and endocrine complications in patients with beta thalassemia major.

*Endocrine complication*							
*Short stature (height centile)*							
Duration of transfusion (years)		<5	5 to 25	25 to 50	>50	Total	P-value
	< 15	59	20	4	1	84	0.29
	≥ 15	41	7	1	2	51	
	Total	100	27	5	3	135	
*Thyroid Function*							
		*Euthyroidism*	*Hypothyroidism*	*Subclinical hypothyroidism*		*Total*	*P-value*
Duration of transfusion (years)	< 15	21	7	1		29	0.27
	≥ 15	21	4	4		29	
	Total	42	11	5		58	
*Parathyroid Function*							
		*Normal*		*Hypoparathyroidism*		*Total*	*P-value*
Duration of transfusion (years)	< 15	4		0		4	0.03
	≥ 15	3		6		9	
	Total	7		6		13	
*Gonadal function*							
		*Normal puberty*		*Delayed puberty*		*Total*	*P-value*
Duration of transfusion (years)	< 15	6		34		40	<0.0001
	≥ 15	24		27		51	
	Total	30		61		91	
*Diabetes*							
		*Absent*		*Present*		*Total*	*P-value*
Duration of transfusion (years)	< 15	76		8		84	0.45
	≥ 15	44		7		51	
	Total	120		15		135	

**Table-III:** Relationship between compliance to chelation therapy and endocrine complications in patients with beta thalassemia major.

*Endocrine complication*							
*Short stature (height centile)*							
		<5	5 to 25	25 to 50	>50	Total	P-value
Compliance to chelation therapy	Yes	47	9	2	2	60	0.52
	No	53	18	3	1	75	
	Total	100	27	5	3	135	
*Thyroid Function*							
		*Euthyroidism*	*Hypothyroidism*	*Subclinical hypothyroidism*		*Total*	*P-value*
Compliance to chelation therapy	Yes	24	6	3		33	0.98
	No	18	5	2		25	
	Total	42	11	5		58	
*Parathyroid Function*							
		*Normal*		*Hypoparathyroidism*		*Total*	*P-value*
Compliance to chelation therapy	Yes	6		4		10	0.42
	No	1		2		3	
	Total	7		6		13	
*Gonadal function*							
		*Normal puberty*		*Delayed puberty*		*Total*	*P-value*
Compliance to chelation therapy	Yes	16		32		48	0.02
	No	14		29		43	
	Total	30		61		91	
*Diabetes*							
		*Absent*		*Present*		*Total*	*P-value*
Compliance to chelation therapy	Yes	53		7		60	0.85
	No	67		8		75	
	Total	120		15		135	

## DISCUSSION

Around 74.1% (128/135) of the study participants suffered from at least one endocrine complication. This indicates a high burden of endocrinopathies amongst patients of BTM. Short stature was the most common endocrinopathy in the study participants with a frequency of 74.1 %, followed by delayed puberty which was seen in 67.03% of the study population. These are very similar to the findings of a study conducted in Karachi which showed that 75 % and 70.8% of the participants had short stature and delayed puberty respectively.^9^ A systematic review conducted in 2021 on short stature in BTM patients showed that 48.9% patients had short stature.^10^ Similarly, a study done in Indonesia in 2018 showed that 60.7% patients had short stature while our study showed 70% patients were below 5^th^ centile on a CDC growth chart. Another study was performed in Iran in 2019 which showed that the prevalence of short stature in BTM patients was 72.6%.^10^-^12^ One of the possible reasons for higher percentage of short stature in our study compared to the above-mentioned studies could be that the criteria of short stature selected by these studies was less than 3^rd^ centile whereas the criteria selected in our study was that of less than 5^th^ centile.

Regarding Thyroid and Para thyroid dysfunction, a study conducted in 2020 revealed hypothyroidism in 26.8% of the participants, while our study showed prevalence of thyroid dysfunction of 26.6% including both hypothyroid (19%) & Subclinical Hypothyroid (8.6%).^13^ Another study was done in 2021 which showed 17.4% hypothyroidism in the studied population, which is quite closer to our study’s findings.^14^ Three studies done in 2018, 2019 & 2022 showed prevalence of hyperparathyroidism in BTM patients as 38%, 13.2% & 22.5% respectively, while current study showed 46.2% patients having hyperparathyroidism among those who were assessed. The reason of higher percentage in our study might be small sample size of patients for hyperparathyroidism assessment.^12^,^15^,^16^

Regarding Diabetes mellitus (DM), a study conducted in Iran in 2017 showed prevalence of DM and pre-diabetes as 10% and 58%, respectively.^17^ Two other studies done in 2019 and 2021 showed prevalence of DM as 15.9% and 14.41%, respectively while in the current study, we had 11.1% BTM patients with DM.^12^,^18^ Regarding pubertal development, which was assessed by examination and serum gonadotropins showed prevalence of delay in puberty in 67.03% patients which was closer to the figure found in study, done in 2019 which showed 44.5% patients were having hypogonadism.^12^

In the current study, 74.1% patients have at least one endocrine disorder as compared to a study done in Iran in 2019 which demonstrated that 86.3% patients have at least one endocrinopathy.^12^ Both of these studies indicate the huge prevalence of endocrinopathies in BTM patients. Regarding knowledge about importance of chelation and keeping ferritin in control range to prevent long term complications, only 3.7% patients were aware of it which is very similar to the study done in Lahore which showed knowledge and awareness in 1.9% patients.^19^

### Limitations:

Although this study exclusively studied patients with BTM for endocrine complications, it has few limitations. Firstly, this study has the weakness of a cross-sectional study where definitive conclusion between the associations and correlations are difficult to obtain. Secondly, this study was conducted at a single center in a tertiary care hospital and the results of this study cannot be generalized unless confirmed by multicenter studies performed at large scale with a larger sample size. Thirdly, certain investigation like growth hormones, IGF1 and estradiol were not done because of financial constraints and were not covered in Sehat Sahulat (insurance card). Finally, only 58 and 13 patients were assessed for thyroid and parathyroid dysfunction respectively because of insurance coverage limitations. Despite these limitations, more than 50% of patients had one or more endocrine complication.

## CONCLUSIONS

This study revealed a high percentage of endocrine complications in patients with BTM. Severity and multiplicity of endocrine organs involvement was dependent on duration of the disease and lack of compliance with chelation therapy. This emphasizes upon the timely and routine screening of these patients for endocrinopathies, and other complications and this standard of care should be adopted by all those centers caring for and treating patients with BTM.
